# Both COVID-19 infection and vaccination induce high-affinity cross-clade responses to SARS-CoV-2 variants

**DOI:** 10.1016/j.isci.2022.104766

**Published:** 2022-07-16

**Authors:** Marc Emmenegger, Sebastian Fiedler, Silvio D. Brugger, Sean R.A. Devenish, Alexey S. Morgunov, Alison Ilsley, Francesco Ricci, Anisa Y. Malik, Thomas Scheier, Leyla Batkitar, Lidia Madrigal, Marco Rossi, Georg Meisl, Andrew K. Lynn, Lanja Saleh, Arnold von Eckardstein, Tuomas P.J. Knowles, Adriano Aguzzi

**Affiliations:** 1 Institute of Neuropathology, University of Zurich, 8091 Zurich, Switzerland; 2 Fluidic Analytics, Unit A, The Paddocks Business Centre, Cherry Hinton Road, Cambridge CB1 8DH, UK; 3 Department of Infectious Diseases and Hospital Epidemiology, University Hospital Zurich, University of Zurich, Zurich, Switzerland; 4 Centre for Misfolding Diseases, Yusuf Hamied Department of Chemistry, University of Cambridge, Lensfield Road, Cambridge CB2 1EW, UK; 5 Department of Laboratory Medicine, University Hospital Zürich, 8091 Zurich, Switzerland; 6 Cavendish Laboratory, Department of Physics, University of Cambridge, JJ Thomson Ave, Cambridge CB3 0HE, UK

**Keywords:** Disease, Immune response, Virology

## Abstract

The B.1.1.529 (omicron) variant has rapidly supplanted most other SARS-CoV-2 variants. Using microfluidics-based antibody affinity profiling (MAAP), we have characterized affinity and IgG concentration in the plasma of 39 individuals with multiple trajectories of SARS-CoV-2 infection and/or vaccination. Antibody affinity was similar against the wild-type, delta, and omicron variants (*K*_A_ ranges: 122 ± 155, 159 ± 148, 211 ± 307 μM^-1^, respectively), indicating a surprisingly broad and mature cross-clade immune response. Postinfectious and vaccinated subjects showed different IgG profiles, with IgG3 (p-value = 0.002) against spike being more prominent in the former group. Lastly, we found that the ELISA titers correlated linearly with measured concentrations (R = 0.72) but not with affinity (R = 0.29). These findings suggest that the wild-type and delta spike induce a polyclonal immune response capable of binding the omicron spike with similar affinity. Changes in titers were primarily driven by antibody concentration, suggesting that B-cell expansion, rather than affinity maturation, dominated the response after infection or vaccination.

## Highlights

• We observe similar antibody affinities against multiple SARS-CoV-2 VOCs
• The antibody profiles show slight differences post-infection versus post-vaccination
• ELISA titers correlate linearly with concentration but not with antibody affinity
• The immune response after SARS-CoV-2 exposure is driven by B cell expansion

## Graphical abstract

**Figure:**
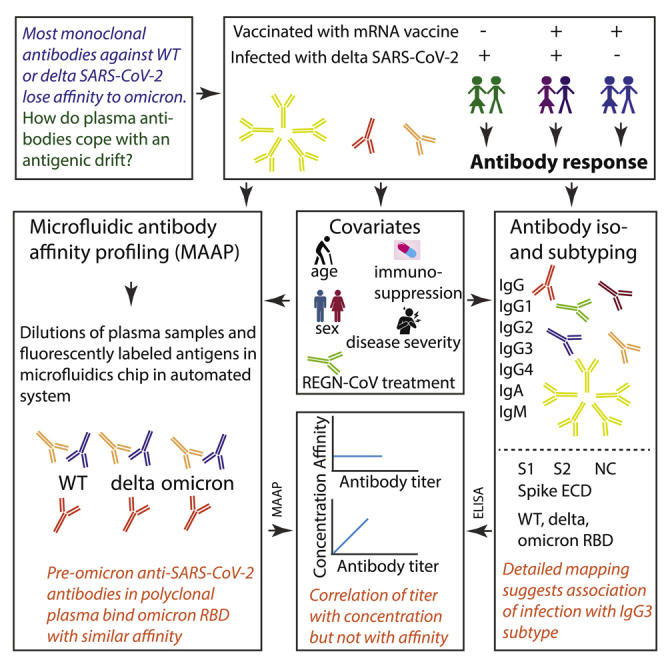

## Introduction

The SARS-CoV-2 B.1.1.529 variant (omicron), considered a WHO variant of concern (VOC) owing to its high transmission rate and its large number of mutations (Han et al., 2022), has become the predominant viral lineage across the globe in early 2022. Its 34 mutations in the spike protein, 15 of which are located within its receptor-binding domain (RBD), which interacts with ACE2, have been shown to impact neutralization (1) of therapeutic monoclonal antibodies in pseudotyped virus-based assays (Cao et al., 2021; Cele et al., 2022; Planas et al., 2021; VanBlargan et al., 2022), (2) of serum antibodies of convalescent patients infected with previous strains, and (3) of serum antibodies of double-vaccinated individuals who had been vaccinated with BNT162b2 (Pfizer-BioNTech), mRNA-1273 (Moderna), Ad26.COV2.S (Johnson & Johnson), ADZ1222 (Astra Zeneca), Sputnik V, or BBIBP-CorV (Sinopharm) (Planas et al., 2021; Cameroni et al., 2022; Dejnirattisai et al., 2022a; Edara et al., 2022; Liu et al., 2022). Although triple vaccination with BNT162b2 or mRNA-1273 or a combination between infection with WT or delta SARS-CoV-2 followed by vaccination increased neutralizing potency compared to double-vaccinated or convalescent serum, titers were still drastically lower for omicron compared with WT or delta SARS-CoV-2 (Planas et al., 2021; Cameroni et al., 2022; Dejnirattisai et al., 2022a; Edara et al., 2022; Liu et al., 2022).

The antibody response against one or multiple epitopes, elicited upon infection or vaccination is characterized by two properties: affinity and concentration. Those are fundamental, well-defined biophysical parameters; however, until recently it has been challenging to measure them directly in complex heterogeneous mixtures, such as serum or plasma. We have recently employed Microfluidic Antibody Affinity Profiling (MAAP) to simultaneously determine the affinity and concentration of antibodies against wildtype (WT) RBD in convalescent sera (Schneider et al., 2022), to study the antibody-based inhibition of RBD-ACE2 interactions (Fiedler et al., 2021) and to understand memory re-activation and cross-reactivity (Denninger et al., 2021). We have also characterized the affinity of multiple therapeutic antibodies (cilgavimab, tixagevimab, casirivimab, and imdevimab) to the omicron RBD variant (Fiedler et al., 2022). Although most of these antibodies exhibited a striking loss of affinity, a pooled plasma standard of anti-SARS-CoV-2 immunoglobulins (Mattiuzzo et al., 2020) retained substantial cross-reactivity to the omicron spike RBD with only moderately decreased antibody concentration and affinity against the omicron variant (Fiedler et al., 2022).

Here, we determined the antibody fingerprints in 39 pre-omicron and two uninfected/non-vaccinated control patients admitted to the University Hospital Zurich, Switzerland. Patients included in this study had a variety of disease trajectories (including no COVID-19) and had received between zero and three doses of vaccine. Using MAAP, we first analyzed antibody affinity and concentration against WT, delta, and omicron RBD variants. We then assessed the impact of vaccination or infection, alone or in combination, as well as of other parameters such as age or disease severity, to antibody concentration, and affinity. Lastly, we characterized the antibody isotype and subtype compositions against SARS-CoV-2 spike domains and against the nucleocapsid (NC) protein using a miniaturized enzyme-linked immunoassay (ELISA) for SARS-CoV-2 antigens called TRABI (Emmenegger et al., 2020, 2021). We found that the natural humoral responses of pre-omicron patients showed less severe reductions of antibody affinity than was observed with monoclonal antibodies and we speculate that this is owing to the polyclonal nature of the infection- or vaccination-induced humoral immune response. Additionally, we found that ELISA-based antibody titers correlated with IgG concentrations but not with affinity, and the antibody profiles in vaccinated but non-infected patients were different from those of patients with a history of SARS-CoV-2 infection.

## Results

### Study design and experimental approach

We have recently described a powerful microfluidics-based technology that enables the affinity determination of complex antibody mixtures in solution in plasma samples (Schneider et al., 2022). Our finding that the affinity of therapeutic monoclonal antibodies is markedly decreased against the SARS-CoV-2 omicron RBD variant (Fiedler et al., 2022) prompted us to investigate the anti-omicron affinity of serum responses in patients who had suffered from pre-omicron COVID-19 or were vaccinated with pre-omicron vaccines.

**Figure 1:**
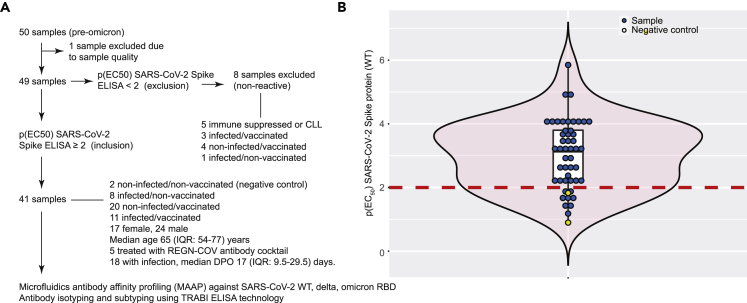
Study design and experimental approach (A) Flowchart for inclusion and exclusion into the study. 41 samples were included in the analysis, representing different patient groups. (B) Violin boxplot showing the distribution of IgG p(EC_50_) values against the SARS-CoV-2 spike protein. A cutoff value of p(EC_50_) ≥ 2 was chosen to define reactive samples. Blue dots represent samples of infected and/or vaccinated individuals. Yellow dots are non-infected and non-vaccinated negative controls.

We collected heparin plasma samples of 50 individuals (pre-omicron) admitted to our hospital. One sample was hemolytic and was excluded from further analyses (Figure 1A). Forty-nine samples were tested for IgG reactivity against the SARS-CoV-2 WT spike protein using the TRABI technology (Emmenegger et al., 2020, 2021). We set a cutoff of p(EC_50_) ≥ 2 for inclusion in subsequent analyses. Eight samples did not reach this threshold and were excluded (Figure 1B). The remaining 41 samples comprised two patients without prior infection/vaccination, eight patients who had suffered SARS-CoV-2 infection but had not received any vaccination, 20 patients who had never been infected with SARS-CoV-2 but received vaccinations (BNT162b2 or mRNA-1273) and 11 patients with previous SARS-CoV-2 infection and vaccination (see Table 1). The presence of infection, prior, or at the time of sampling, was inferred based on medical history and/or one or multiple positive SARS-CoV-2 RT-qPCR. The median age of enrolled patients was 65 (interquartile range (IQR): 54–77) years. Among the patients with a history of infection (n = 19), the median days post-onset of disease manifestation (DPO) was 12 (IQR: 8.25–17.75) days and for those individuals with a history of vaccination, the median days post last vaccination (DPV) was 176 (48–238) days. For five patients whose infection dated back more than a month before sampling, the exact DPO could not be inferred from the clinical record. These samples were analyzed using MAAP (Denninger et al., 2021; Fiedler et al., 2022; Schneider et al., 2022) with SARS-CoV-2 WT, delta, and omicron RBD variants. Antibody isotypes and subtypes were further assessed in the same samples using TRABI (Emmenegger et al., 2020, 2021).

**Table 1:** Aggregated characteristics of individuals included in the study

	n	female	male	age (median, IQR), years	REGN-COV-treated	DPO (MEDIAN, IQR), days	DPV (MEDIAN, IQR), days
all individuals	41	17	24	65, 54–77	5	12, 8.25–17.75	176, 48–238
Non-infected/non-vaccinated	2	1	1	58.5, 58.25–58.75	0	–	–
Infected/non-vaccinated	8	4	4	61, 53–66	1	16, 13.5–23	–
Non-infected/vaccinated	20	8	12	63, 57–78	0	–	159.5, 39.5–250.25
Infected/vaccinated	11	4	7	74, 61–84	4	9.5, 7.5–12.75	196, 76–212

DPO = day post onset of COVID-19 symptoms. DPV = day post most recent vaccination.

### Characterization of affinity of SARS-CoV-2 antibodies to wildtype, delta, and omicron receptor-binding domain variants

**Figure 2:**
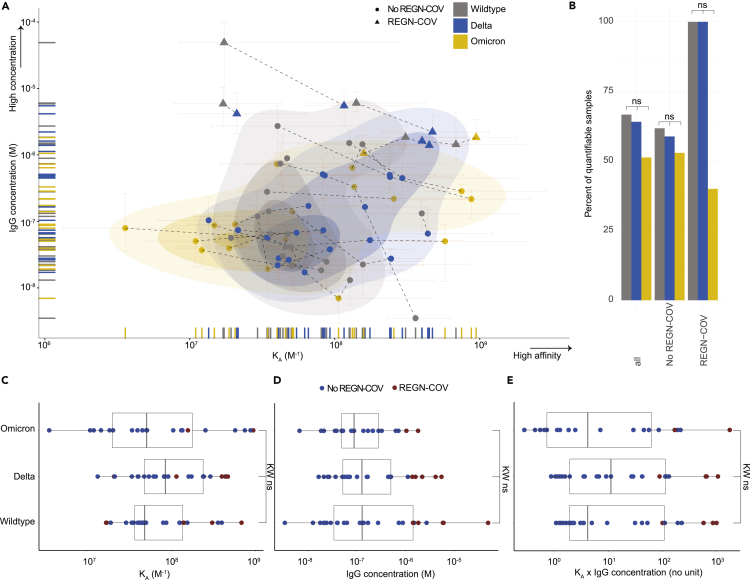
Characterization of affinity of SARS-CoV-2 antibodies to WT, delta, and omicron RBD variants (A) 2D scatter plot with integrated density contours. All quantifiable data points reflecting *K*_A_ (in M^−1^) and IgG concentration values (in M) are plotted. 95% confidence intervals for each point are colored in light red. Triangles denote patients receiving the REGN-COV cocktail. RBD variants: WT (grey), delta (blue), omicron (yellow). Dotted lines represent the measurements of the same patient sample against different RBD variants. (B) Bar graph displaying the percentages of quantifiable samples for WT (grey), delta (blue), and omicron (yellow) RBD variants. Comparisons were performed including all samples, samples excluding those treated with REGN-COV, and only those treated with REGN-COV. Fisher’s exact test displayed no significant differences, at *α* = 0.01. (C and D) Boxplot analysis of *K*_A_ values (C) and IgG concentrations (D) for WT, delta, and omicron RBD variants. (E) To employ a combined score of binding affinity (*K*_A_) and IgG concentration, we utilized the product *K*_A_ x IgG concentration. (C-E): Colors denote treatment with REGN-COV (red) or absence of treatment (blue). Kruskal-Wallis (KW) with post-hoc Wilcoxon rank sum test (WC) after Holm correction for multiple comparisons was used, with *α* = 0.01. None of the group-wise comparisons reached statistical significance.

We measured the affinities and concentrations of patient samples to WT, delta, and omicron RBD. We report the affinity constant (*K*_A_), which is 1/*K*_D_, where *K*_D_ is the equilibrium dissociation constant. Measured antibody affinity constants ranged between 3.59 μM^-1^ (*K*_D_: 278 nM, i.e. comparatively low affinity) and 943.3 μM^-1^ (*K*_D_: 1 nM, i.e. comparatively high affinity). Thus, we observed an approximately 250-fold affinity range within our cohort. The IgG concentrations varied between 3 and 49,074 nM (range: 4.2 log_10_) (Figure 2A). The integrated 2D-density plot revealed a moderate left shift, i.e. overall decreased *K*_A_, of antibodies to omicron while none of the variants formed separate clusters and mostly overlapped. 49% of samples could not be quantified in terms of *K*_A_ or IgG concentration for omicron (33% for wildtype and 36% for delta) (Figure 2B), indicating that the affinity or concentration was outside the sensitivity of the assay. Using a dataset of COVID-19 convalescent donors and Alexa 647-labeled RBD (Fiedler et al., 2022), MAAP enabled a reliable measurement up to approximately 100 nM (*K*_*D*_, see Figure S1A), i.e. down to 0.01 nM^−1^ (*K*_*A*_, see Figure S1B) and concentrations as low as 10 nM (Figure S1C). Although an increase in serum fraction typically enhances the availability of antibodies in samples with low concentration and/or low affinity, the lower limit of the labeled antigen concentration that can be employed is reduced owing to intrinsic background fluorescence, which may compromise the sensitivity of the assay (see Figure S1D). The number of non-quantifiable samples reported here reflects the fraction of samples where no value was obtained, owing to concentrations below 10 nM or *K*_D_ values clearly above the range of 100 nM or owing to a combination of both.

In our dataset, the distributional differences of quantifiable/non-quantifiable samples did not significantly differ (Fisher’s exact test, *α* = 0.01, Figure 2B) among any of the antigens, for all samples (n = 39), for those with an exclusively infection- and/or vaccine-induced antibody response (n = 34), or for those treated with the REGN-COV cocktail (casirivimab and imdevimab, n = 5), although a trend towards increased evasion of antibody binding for omicron was visible.

We then investigated *K*_A_ (Figure 2C), IgG concentrations (Figure 2D) and the product of *K*_A_ x IgG concentration (Figure 2E) for the three RBD variants. None of the variants displayed a statistically significant deviation (Kruskal-Wallis with post-hoc Wilcoxon rank sum test after Holm correction for multiple comparisons, with *α* = 0.01). Five patients who received the REGN-COV antibody cocktail displayed the highest IgG concentrations measured, yet their affinities were in the range of the non-REGN-COV-treated patients (Figures 2A and 2C–2E). In sum, the antibody response following infection and/or vaccination appears less susceptible to a drastic loss in binding against the omicron variant compared to monoclonal antibodies.

### Correlation of antibody fingerprints with clinically relevant parameters does not reveal clear differences between vaccinated and infected subgroups

**Figure 3:**
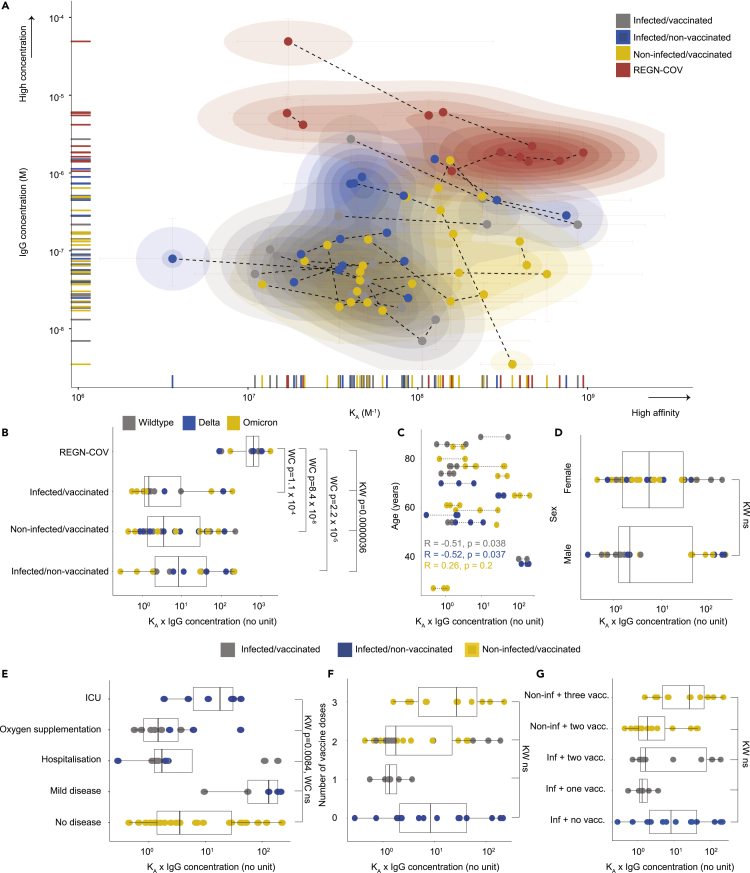
Correlation of affinity and IgG concentrations with clinically relevant parameters does not reveal clear differences between vaccinated and infected subgroups (A) 2D scatter plot with integrated density contours. All quantifiable data points reflecting *K*_A_ (in M^−1^) and IgG concentration (in M) are plotted. 95% confidence intervals for each point are colored in light red. No distinct clusters were observed among patient groups infected/vaccinated (grey), infected/non-vaccinated (blue), non-infected/vaccinated (yellow); however, the REGN-COV-treated patients (red) clustered separately. (B) The same groups as in (A) depicted in a boxplot. Statistical analysis is shown in the graph. The RBD variants are color-coded. (C and D) No correlation between age (C) or sex (D) and *K*_A_ x IgG concentration. (E) Although Kruskal-Wallis statistical testing indicates that the distributions are significantly different for different disease severities, pair-wise testing with the Wilcoxon rank sum test does not result in significance. (F) Trend towards increased *K*_A_ x IgG concentration products in triple vaccinated individuals, without being statistically significant. (G) Same as (F) but additionally stratified according to vaccination/non-vaccination. A: Dotted lines represent the measurements of the same patient sample against different RBD variants. B, D-G: Kruskal-Wallis (KW) with post-hoc Wilcoxon rank sum test (WC) after Holm correction for multiple comparisons was used, with α = 0.01. C: The Pearson correlation coefficient was calculated. C-G: The patient groups are color-coded as in (A); however, the REGN-COV-treated patients were excluded from analyses.

We next characterized the affinity/concentration profiles in four patient groups: (1) infected/non-vaccinated; (2) non-infected/vaccinated; (3) infected/vaccinated; (4) treated with REGN-COV. We studied the same profile as above, but we color-coded the data points according to the groups of patients (Figure 3A). The patients treated with the REGN-COV cocktail clustered separately, as expected, whereas the density representations for vaccinated and/or infected patient groups were largely overlapping. Statistical testing showed that the *K*_A_ x IgG concentration product significantly differed in patients treated with the REGN-COV cocktail versus all other groups (Wilcoxon rank sum test after Holm correction, Figure 3B). However, the profiles observed following vaccination and/or infection did not statistically differ among each other. In the following analyses, we have focused solely on those groups with physiological antibody responses and excluded the REGN-COV-treated patients. Correlations of *K*_A_ x IgG concentration with age (Figure 3C, the Pearson correlation coefficient R was calculated for the three groups), sex (Figure 3D), or with disease severity (Figure 3E) revealed heterogeneity rather than marked differences. The Kruskal-Wallis test indicated significant distributional differences as a function of disease severity (p-value = 0.0084), yet, pair-wise testing with Wilcoxon rank sum test did not result in any significantly changed group after correcting for multiple comparisons. Although we observed a trend towards increased *K*_A_ x IgG concentration with a higher number of vaccinations (Figure 3F), the distributions did not significantly differ. Accordingly, three vaccinations, compared with two, lead to a marked increase among the non-infected population, whereas two vaccinations, compared with one, lead to a slight increase in the infected population (Figure 3G). However, Kruskal-Wallis test indicated no significant differences among all groups (p-value = 0.022).

While larger samples may display the expected distributional shift towards higher concentrations and/or affinity after multiple boosters, the combinations of infections and vaccinations do not exert a measurable effect on *K*_A_ and IgG concentrations in the complex samples used in this study.

### Analysis of antibody subtypes, correlation with affinity, and global feature profiling

As infection, vaccination, or parameters such as disease severity, age, and number of vaccinations did not clearly correlate with antibody affinity or concentration, we aimed to obtain a more granular view of the antibody compositions. Thus, we used TRABI (Emmenegger et al., 2020, 2021) to deeply characterize the antibody iso- and subtypes in our patient collective, to compare it to antibody affinity and concentration, and to seek potential clinical or demographic correlates.

**Figure 4:**
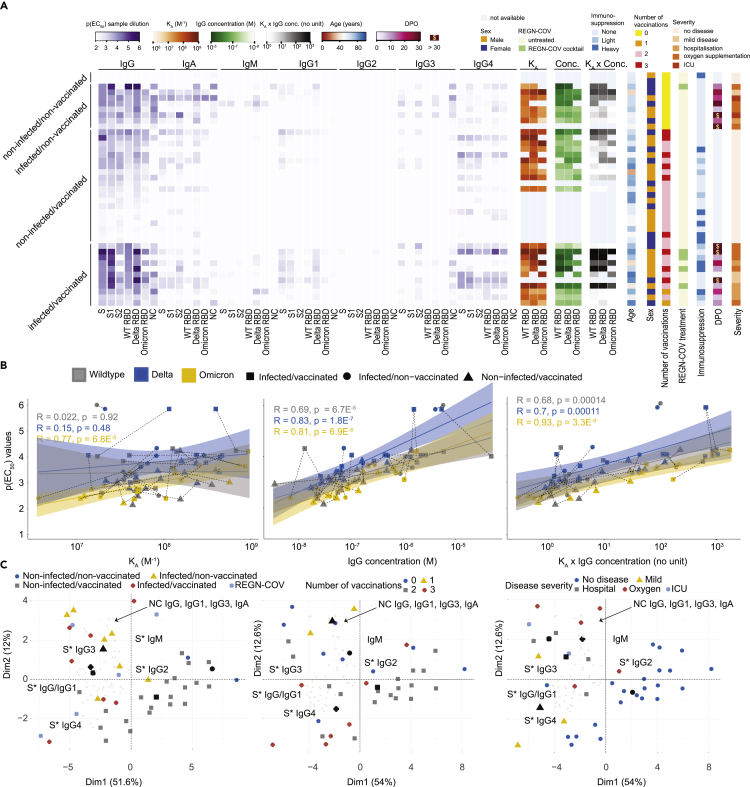
Analysis of antibody subtypes, correlation with MAAP parameters, and global feature profiling (A) Multiple heatmaps. Purple heatmap displaying p(EC_50_) values (gradient) obtained with TRABI ELISA, for IgG, IgA, IgM, IgG1, IgG2, IgG3, and IgG4 antibodies. The SARS-CoV-2 WT spike ectodomain, the WT S1 domain, the WT S2 domain, the WT RBD, the delta RBD, and the omicron RBD variants as well as the nucleocapsid (NC) proteins were used. Orange heatmap displaying *K*_A_ values, green heatmap displaying IgG concentration, grey heatmap displaying *K*_*A*_ x IgG concentration obtained with MAAP against WT, delta, and omicron RBD variants. Additional heatmaps depict the age (red to blue), sex (orange: male; blue: female), number of vaccinations (yellow = 0, orange = 1, purple = 2, red = 3), treatment with the REGN-COV cocktail (green = TRUE), the strength of immunosuppression (none = light blue, light = turquoise, heavy = dark blue), the days post onset of infection (DPO) for patients with infection (pink), and disease severity (orange gradient). (B) Correlation between IgG p(EC_50_) values of the spike ectodomain with *K*_A_, IgG concentrations, or the product *K*_A_ x IgG concentration. (C) Principal component analysis using all TRABI ELISA values as input. The three plots represent different color-based clustering approaches.

We first measured IgG, IgA, IgM, IgG1, IgG2, IgG3, and IgG4 antibodies against the SARS-CoV-2 WT spike ectodomain (ECD), the WT S1 domain, the WT S2 domain, the WT RBD, the delta RBD, and the omicron RBD variants as well as the nucleocapsid (NC) proteins and illustrated them in a heatmap (Figure 4A, purple gradients). The antibody profile mainly revealed that the antibody response, in general, is dominated by IgG, followed by IgA and much less so by IgM and that all IgG subtypes, except IgG2, contributed to the IgG response against the spike-associated domains (Figure S2A). The presence of IgG antibodies against the NC, the only protein employed here that is not intrinsically connected to the spike ECD, is indicative of an infection, which was observed in almost all patients with clinically characterized infection with SARS-CoV-2. For NC, the dominant IgG subtype was IgG3 (Figure S2B).

To validate our results, we repeated the IgG4 measurements against the entire collection of antigens, with the same IgG4-specific secondary antibody (Figure S2C) and with the same clone but different storage buffer sold by a different vendor (Figure S2D). We observed robust correlations using the same IgG4-specific secondary antibody (Pearson correlation coefficient R = 0.94) as well as the same antibody clone from a different source (Pearson correlation coefficient R = 0.93) in three fully independent experiments. The specificity of the secondary antibodies in detecting the desired immunoglobulin iso- and subtypes was extensively validated (Figure S3). Moreover, the IgG titers measured for the three RBD variants via ELISA correlated well among each other (Figures S4A–S4C) and with the spike protein (Figure S4D) Similarly, the immunoglobulin iso- and subtype compositions have been largely congruent for the three RBD variants, as shown using the mean p(EC_50_) values (Figure S4E).

We then included additional features such as the *K*_A_ values, the IgG concentrations, age, sex, indications for treatment with REGN-COV antibodies, immunosuppression (none, light, heavy), the DPO as well as disease severity, and aligned these values for each patient, separated into the vaccination/infection groups (Figure 4A and Table S1). This view offers a comprehensive multidimensional assessment of many parameters at the single-individual level. We first correlated the IgG ELISA p(EC_50_) values obtained against WT, delta, and omicron RBD with the respective MAAP-derived *K*_A_, the IgG concentration, and the product *K*_A_ x IgG concentration (Figures 4B and S5 for a general representation). Although *K*_A_ showed no linear relationship with ELISA titers (average R over all groups = 0.29, p-value = 0.015), both IgG concentration (average R over all groups = 0.72, p-value = 5.4 × 10^−13^) as well as the MAAP product (average R over all groups = 0.71, p-value = 5.4 × 10^−12^) were well represented by a linear model, for all the three variants. This finding suggested that the titers observed in ELISA primarily reflect antibody concentrations rather than affinities in samples analyzed here.

We then reduced the dimensionality across all measured antibodies using principal component analysis (PCA) and projected the linear combinations in two-dimensional space (Figures 4C and S6 for a granular view on the variable map). We used three representations using colors and shapes: (1) The infection/vaccination cohorts, where we included patients treated with the REGN-COV antibodies, (2) the number of vaccinations (excluding REGN-COV), (3) disease severity (excluding REGN-COV). Regional clusters of specific antibody iso- and subtypes were annotated in black. Black shapes indicate the mean points of a given group (indicated by color and shape). PCA suggested that while patients with infection (infected/non-vaccinated; infection/vaccinated) clustered towards spike-associated (annotated as S∗) IgG3 as well as NC IgG, IgG1, IgG3, and IgA, patients with vaccination (non-infected/vaccinated: infected/vaccinated) clustered towards spike-associated IgG4. Spike-associated IgG, as well as IgG1, were between the two groups. Moreover, a higher number of vaccinations (two or three) appeared to be linked to spike-associated IgG4 positivity, while fewer vaccinations (none or one) clustered more closely to spike-associated IgG3 as well as to NC IgG, IgG1, IgG3, and IgA. A largely similar pattern was observed with disease severity. No or mild disease clustered more in the region of spike-associated IgG4 while more severe disease courses (hospitalization, oxygen supplementation, ICU) assembled in the region of spike-associated IgG3 and the NC sub- and isotypes referred to above. In sum, this representation evidenced an association between infection, more severe disease, absence of vaccinations, and an IgG3 response against the spike-associated proteins. Conversely, the IgG4 response against spike-associated proteins was mainly characterized by vaccination, with higher repeats of vaccinations, and a less severe disease course on average.

### Multidimensional analysis suggests slightly different antibody profiles in patients after SARS-CoV-2 infection versus vaccination alone

**Figure 5:**
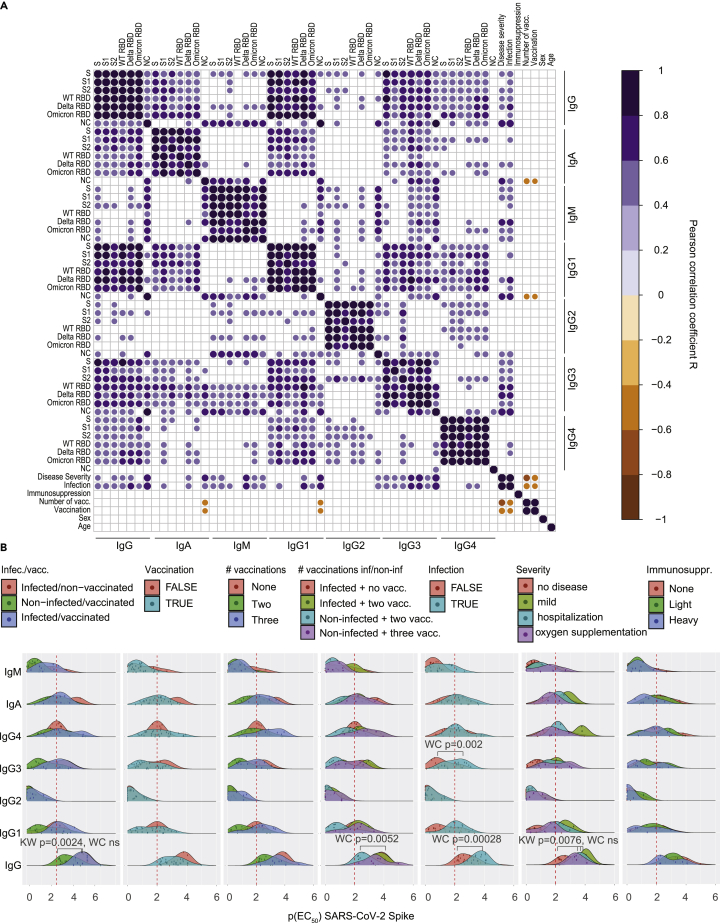
Multidimensional analysis of antibody profiles in patients after SARS-CoV-2 infection versus vaccination (A) Correlogram analysis using the TRABI ELISA values combined with features such as disease severity, immunosuppression, number of vaccinations received, sex, and age. Only significant correlations are shown, at *α* = 0.01. (B) Ridge plot distributions of p(EC_50_) values for all immunoglobulin iso- and subtypes against the Spike ECD. Data were aggregated according to the patient group, vaccination, number of vaccinations, infection, disease severity, and immunosuppression. Kruskal-Wallis (KW) with post-hoc Wilcoxon rank sum test (WC) after Holm correction for multiple comparisons was used, with *α* = 0.01. Only significant changes are displayed.

Based on the patterns identified above, we analyzed potential associations using different methods. We first calculated the Pearson correlation coefficients for all antigens and antibody iso- and subtypes and included additional parameters such as disease severity, immunosuppression, the REGN-COV cocktail, the number of vaccinations, sex, and age, and plotted the significant correlations in a correlogram (Figure 5A). Globally, the correlogram indicated a pronounced positive correlation within the iso- or subtypes, which is expected as all domains are contained within the spike ECD, except the NC antigen. IgG1 correlated almost perfectly with IgG, while IgM and IgG2 displayed only spurious correlations with IgG. Disease severity correlated with reactivity against the NC protein, for IgG, IgA, IgG1, and foremostly IgG3 but not for IgG2 or IgG4. A higher number of vaccinations showed negative correlations with NC for IgA and IgG1. Sex and age did not display strong correlations in any direction. The same correlogram with all correlations irrespectively of the significance level is shown in Figure S7.

We then looked into the antibody reactivity profiles that have shown relevance based on the PCA representation and the correlogram. The p(EC_50_) values of seven Ig iso- and subtypes for the SARS-CoV-2 spike ECD were represented as a ridge plot. We focused only on the spike protein as a well-correlated surrogate for other spike domains (see Figure S3D), and omitted the NC protein from this analysis. We compared the groups per antibody iso- and subtype looking at the entire distribution by means of Kruskal-Wallis test with post-hoc Wilcoxon rank sum test corrected for multiple comparisons with Holm (*α* = 0.01). Exclusively significant distributional differences were annotated the in plot (see Figure 5B). Kruskal-Wallis statistics indicated that the IgG responses of the non-infected/vaccinated groups showed a significant change among each other (p-value = 0.0024); however, the group-wise testing by means of the Wilcoxon rank sum test did not reach statistical significance owing to multiple comparisons that were performed. Likewise, the IgG distributions differed among the degrees of severity (p-value = 0.0076, Kruskal-Wallis), but pair-wise testing resulted in non-significant differences. The IgG4, but not overall IgG, IgG1, IgG2, or IgG3 distributions of patients after vaccination showed a trend towards higher p(EC_50_) values, which were increased after three vaccinations. Yet, this observation did not reach statistical significance. However, infection was associated with higher p(EC_50_) values for IgG (p-value = 0.00028, Wilcoxon rank sum test after Holm correction), and IgG3 (p-value = 0.002, Wilcoxon) but not for IgG1, IgG2 or IgG4. In line with this, infection without vaccination led to significantly increased IgG, but not IgG1, IgG2, IgG3, IgG4, IgA, or IGM, levels compared with (1) the non-infected group after two rounds of vaccination (p-value = 0.0052, Wilcoxon) but was not different compared with (2) infection with two vaccinations or with (3) three vaccinations in the absence of prior infection. Overall, the available evidence in this heterogeneous cohort used for data mining thus points towards slight alterations in the profiles following vaccination or infection. Yet, immunoglobulin profiles are subject to temporal dynamics and larger cohorts may be required to substantiate the findings from our broad explorative approach.

## Discussion

We reported that the monoclonal antibodies deployed against previous variants of SARS-CoV-2 show drastically reduced affinity against the omicron variant (Fiedler et al., 2022). Here, we found that delta-infected and/or vaccinated subjects develop plasma antibodies with similar affinities to all major SARS-CoV-2 clades. Previous SARS-CoV-2 infection (alone or in combination with vaccination), but not vaccination alone, was associated with higher overall anti-SARS-CoV-2 spike IgG, and IgG3 titers. Thus, although antibody profiles following infection differ from that of vaccinated patients who did not encounter the virus, pre-omicron responses showed impressive cross-clade affinities.

The high-affinity responses to the omicron spike protein contrast starkly with those of current therapeutic monoclonal antibodies (Fiedler et al., 2022) whose the neutralization of omicron was poor or absent (Dejnirattisai et al., 2022b; Planas et al., 2021; VanBlargan et al., 2022). The *K*_A_ of plasma antibodies against SARS-CoV-2 RBD variants were in a similar range of 16.9–684.9 μM^-1^ for WT, 13.5–471.7 μM^-1^ for delta, and 3.6–943.4 μM^−1^ for omicron. These data may provide an explanation for the observation that patient sera after three doses of the Pfizer-BioNTech mRNA vaccine and convalescent individuals after a Pfizer-BioNTech booster retained neutralization capacity against omicron (Planas et al., 2021). In line with our observation, the RBD-specific memory B-cell repertoire was shown to diversify after the second vaccination, and to expand and generate even more clones after a third vaccination, which increases the breadth and capability of the immune system to respond to SARS-CoV-2 VOC (Muecksch et al., 2022). Thus, whereas monoclonal antibodies, which are typically selected *in vitro* for the smallest possible dissociation constant to the constituents of a given VOC, are prone to lose their binding properties when confronted with another VOC, the polyclonality of the natural immune response can plausibly neutralize not only a given pathogen but also variations thereof.

As expected, the spike-specific IgG concentrations of the REGN-COV cocktail administered to patients exceeded the IgG concentrations following a genuine immune response triggered by infection or vaccination by approximately 30-fold and the *K*_A_ x IgG concentration product was significantly different in the group treated with REGN-COV versus all other groups. However, we did not identify any significant correlation between *K*_A_ or IgG concentrations and parameters such as infection or vaccination, alone or in combination, the severity of disease, or with sex and age. Although the number of vaccinations has not shown significant differences in given, clearly limited, sample size, the trend towards increased *K*_A_ x IgG concentration scores as a function of repeated vaccination was evident.

To increase the depth of our data set, we added data complementary to antibody affinity and concentrations and mapped the contributions of different iso- and subtypes measured by TRABI ELISA (Emmenegger et al., 2020, 2021), using a comprehensive panel of antigens (WT spike ectodomain, WT spike S1, WT spike S2, WT RBD, delta RBD, omicron RBD, NC protein). We observed that the p(EC_50_) values of the RBD variants derived by ELISA correlated well with each other, in line with affinity measurements, as well as with the WT spike. However, while ELISA titers correlated poorly with affinity measurements, they were in excellent agreement with the IgG concentrations. Hence antibody titers measured in ELISA, typically a conflation of both affinity and concentration, were mostly driven by concentrations rather than affinities. Most likely this is because at an estimated ELISA plate surface-bound RBD concentration of about 480 nM, the expected [antibody-RBD] complex concentration is much more sensitive to antibody concentration than to antibody affinity, provided that antibody concentrations exceed *K*_D_. At low affinities (high *K*_D_ values) close to the measured antibody concentration or if the antibody concentrations drop to about the *K*_D_ or lower, the [antibody-RBD] complex concentration becomes much more sensitive to affinity. At a mean *K*_D_ of 6 nM and IgG concentration of 1 μM in our dataset (across all RBD variants measured), our immobilization-based ELISA measurements are thus mostly influenced by antibody concentration.

Several patients had IgG4 antibody titers against spike domains, but not against the NC, a phenomenon that had not been reported to the same extent (Amanat et al., 2020; Suthar et al., 2020; Klingler et al., 2021; Kober et al., 2022; Sievers et al., 2022) although IgG4 antibodies against spike domains have been described (Farkash et al., 2021; Jarlhelt et al., 2021). Our observation was corroborated by repeating the measurements using the same IgG4-specific secondary antibody as well as by using the same clone from an alternative source (including differences in concentrations and storage buffers), which makes us confident that the results presented are valid. Moreover, we presented one of the most comprehensive cross-validations to date of all secondary antibodies – widely used in research on SARS-CoV-2 (Crowley et al., 2021; Fraley et al., 2021; Kim et al., 2021; Lee et al., 2021; Newell et al., 2021; Phelan et al., 2021; Pullen et al., 2021; Yates et al., 2021) and in other fields (Minassian et al., 2021; Sanchez Vargas et al., 2021; Tschismarov et al., 2021; Jennewein et al., 2022; Toney et al., 2022) – employed in this study and have confirmed their specificity to their target. We have noted that the sensitivity of the anti-human IgG1 antibody used can benefit from a higher concentration; however, it would come at a cost of compromising its specificity as the antibody shows cross-reactivity to other IgG subtypes at slightly increased concentrations, therefore, presenting a less favorable sensitivity-to-specificity profile than the other secondary antibodies. The surprisingly higher prevalence of the IgG4 subtype might arise owing to repeated encounters with the antigen, which earlier studies on COVID-19 may not have captured. In this context, repeated infections have previously been described to elevate total IgG4 levels (Ebbo et al., 2012).

We next explored the antibody profiles using a feature-based dimensionality reduction and comprehensive correlograms. Both approaches pointed towards a slightly altered antibody profile following vaccination or infection, with a stronger IgG3 response upon infection. However, the clusters were relatively weak and potentially ambiguous in our dataset, although a previous investigation derived similar conclusions regarding differences in the profiles between vaccinated and convalescent individuals (Klingler et al., 2021). Therefore, we focused on WT spike ECD, by looking at the distribution in a statistical manner. We confirmed that infection alone or in combination with vaccination was associated with higher p(EC_50_) values for IgG and IgG3 but not for IgG1, IgG2 or IgG4. These differences may be partly associated with the respective SARS-CoV-2 VOC, as infections with heterologous variants and/or multiple exposures to antigens may alter immune profiles (Reynolds et al., 2022). Furthermore, these profiles underlie different temporal dynamics and might reflect different time points at which the antigen was last encountered. The use of MAAP in larger cohorts may corroborate the observations of the present exploratory study.

In conclusion, we have investigated antibody affinity and concentration following infection and/or vaccination in the presence of antigenic drift. We found that the tolerance to the omicron drift was surprisingly robust, whereas the currently approved therapeutic monoclonal antibodies lost much of their affinity. The most plausible scenario is that antibodies are selected *in vivo* for immunodominant spike domains that are invariant between clades of virus, whereas therapeutic monoclonals were presumably selected *in vitro* for highest affinity but not for cross-clade protection. Ultimately, our finding, along with others, suggests that the B-cell-mediated immunity, possibly concomitant with a T-cell response, elicited upon infection and/or vaccination might be broad enough to confer a layer of protection in the event of further waves of mutated SARS-CoV-2 variants.

### Limitations of the study

The limitations of our investigations reside in the number of patients enrolled in the study and the vast number of variables reported, which may constrain the generalizability of results and conclusions. All data underlying this study will be made available for further studies and for comparison with future cohorts. On the other hand, our findings describing the antibody response of pre-omicron convalescent or post-vaccination sera to the SARS-CoV-2 omicron variant are congruent with those found by others with other methods (see e.g. (He et al., 2022)), including viral neutralization and clinical observations.

## STAR★Methods

### Key resources table

**Table:** 

REAGENT or RESOURCE	SOURCE	IDENTIFIER
**Antibodies**		

Goat anti-human IgG, 1:4000	Jackson	109-035-098; RRID: AB_2337586
Goat anti-human IgA, 1:750	Thermo Fisher Scientific	31417; RRID: AB_228253
Goat anti-human IgM, 1:3,000	Sigma-Aldrich	A6907; RRID: AB_258318
Mouse anti-human IgG1, 1:3,000	SouthernBiotech	9054–05; RRID: AB_2796627
Mouse anti-human IgG2, 1:3,000	SouthernBiotech	9060–05; RRID: AB_2796633
Mouse anti-human IgG3, 1:3,000	SouthernBiotech	9210–05; RRID: AB_2796699
Mouse anti-human IgG4, 1:3,000	SouthernBiotech	9200–05; RRID: AB_2796691
Mouse anti-human IgG4, 1:500	Invitrogen	A-10654; RRID: AB_2534054

Chemicals, Peptides, and Recombinant Proteins		

spike ECD (for ELISA), HEK293, 1 μg/mL	(Emmenegger et al., 2020), Oxford	N/A
WT spike S1 (for ELISA), HEK293, 1 μg/mL	AcroBiosystems	S1N-C52H2
WT spike S2 (for ELISA), HEK293, 1 μg/mL	AcroBiosystems	S2N-C52H5
WT RBD (for ELISA), HEK293, 1 μg/mL	(Emmenegger et al., 2020), Oxford	N/A
WT RBD (for MAAP), HEK293, 1 μg/mL	Sino Biological	40592-V08H
Delta RBD (for ELISA, MAAP), HEK293, 1 μg/mL	Sino Biological	40592-V08H90
Omicron RBD (for ELISA, MAAP), HEK293, 1 μg/mL	Sino Biological	40592-V08H121
NC (for ELISA), HEK293, 1 μg/mL	AcroBiosystems	NUN-C5227
Purified IgA (for ELISA), Human serum, 2.5 μg/mL	Sigma-Aldrich	14036
Purified IgM(for ELISA), Human serum, 2.5 μg/mL	Sigma-Aldrich	18260
Recombinant human IgG1 (for ELISA), HEK293, 2.5 μg/mL	Mabylon AG	N/A
Recombinant human IgG2 (for ELISA), Human myeloma, 2.5 μg/mL	EMD Millipore	AG504; RRID: AB_97839
Recombinant human IgG3 (for ELISA), Human myeloma, 2.5 μg/mL	EMD Millipore	AG506; RRID: AB_97840
Recombinant human IgG4 (for ELISA), HEK293, 2.5 μg/mL	Mabylon AG	N/A

### Resource availability

#### Lead contact

Further information and requests for resources should be directed to and will be fulfilled by the lead contact, Adriano Aguzzi (adriano.aguzzi@usz.ch).

#### Materials availability

Small amounts of the biological samples can be shared if available, upon reasonable request, and if an approval by an ethics committee as well as an MTA is in place.

### Experimental model and subject details

#### Ethics statement

For this study, we included residual pre-omicron heparin plasma samples from patients (median age 65 (interquartile range (IQR): 54–77) years; distribution of female-male sex 0.41:0.59, see Tables 1 and S1) admitted to the University Hospital Zurich, Zurich, Switzerland, whose blood was sent to the Institute of Clinical Chemistry for routine diagnostic procedures. Infections with the SARS-CoV-2 B.1.1.529 variant were excluded by means of dropout PCR. All experiments and analyses involving samples from human donors were conducted with the approval of the ethics committee of the canton Zurich (KEK Zürich), Switzerland (KEK-ZH-Nr. 2015–0561, BASEC-Nr. 2018–01042, and BASEC-Nr. 2020–01731), in accordance with the provisions of the Declaration of Helsinki and the Good Clinical Practice guidelines of the International Conference on Harmonisation. All subjects enrolled in the study signed the hospital-wide General Consent of the University Hospital Zurich, Switzerland.

### Method details

#### Fluorescent labeling of proteins

Recombinant proteins were labeled with Alexa Fluor 647 NHS ester (Thermo Fisher) as described previously (Fiedler et al., 2021, 2022). In brief, solution containing 150 μg of spike RBD was mixed with dye at a three-fold molar excess in the presence of NaHCO3 (Merck) buffer at pH 8.3 and incubated at 4°C overnight. Unbound label was removed by size-exclusion chromatography (SEC) on an ÄKTA pure system (Cytiva) using a Superdex 75 Increase 10/300 column (Cytiva). Labeled and purified proteins were stored at −80°C in PBS pH 7.4 containing 10% (w/v) glycerol as cryoprotectant.

#### Antibody affinity and concentration determination

Microfluidic Antibody Affinity Profiling (MAAP) measurements were performed as reported previously (Schneider et al., 2022). For the MAAP measurements, varying fractions of human plasma samples were added to a solution of the antigen of concentrations varying between 1 nM and 400 nM, and a buffer containing PBS at pH 7.4, 0.05% (w/v) Tween 20 (Merck), 5% (w/v) human serum albumin (Merck), and 10% (w/v) glycerol (Merck). The antigens used were RBD (Sino Biological; WT 40592-V08H, Delta 40592- V08H90, Omicron 40592- V08H121, see Key resources table) labelled with Alexa Fluor^TM^ 647 (Thermo Fisher) through amine coupling. These samples were incubated on ice for 30 min and the size of the formed immunocomplex was determined through measuring the hydrodynamic radius, *R*_h_, with Microfluidic Diffusional Sizing (MDS) using the commercial Fluidity One-M platform. The data were analysed by Bayesian inference as described previously (Linse et al., 2020; Schneider et al., 2022).

#### High-throughput TRABI ELISA

Serological ELISAs were carried out as previously described (Emmenegger et al., 2020, 2021) with minor adjustments. High-binding 1,536-well plates (Perkin-Elmer; SpectraPlate 1536 HB) were coated with 3 μL of 1 μg/mL SARS-CoV-2 spike ECD, WT S1, WT S2, WT RBD, delta RBD, omicron RBD, or NC protein in PBS using Fritz Gyger Certus Flex, incubated at 37 °C for 1 h in a ThermoFisher rotating plate incubator, and washed three times with PBS 0.1% Tween-20 (PBS-T) using Biotek El406. Plates were blocked with 10 μL of 5% milk in PBS-T for 1.5 h using Biotek Multiflo FX peristaltic dispensing technology. Samples inactivated with 1% Triton X-100 and 1% tributyl phosphate were diluted in sample buffer (1% milk in PBS-T), and a serial dilution (range: 0.02 to 1.6 × 10^−4^) was carried out (volume: 3 μL per well) on an ECHO 555 acoustic dispenser (Labcyte) using contactless ultrasound nanodispensing. After the sample incubation for 2 h at RT, the wells were washed five times with wash buffer, and the presence of anti–SARS-CoV-2 antibodies was detected using horseradish peroxidase (HRP)-linked antibodies (1. anti-human IgG antibody: Peroxidase AffiniPure Goat Anti-Human IgG, Fcγ Fragment Specific; Jackson; 109-035-098 at 1:4,000 dilution. 2. anti-human IgA antibody: Goat Anti-Human IgA Heavy Chain Secondary Antibody, HRP; Thermo Fisher Scientific; 31417 at 1:750 dilution. 3. anti-human IgM antibody: anti-human IgM μ-chain–specific antibody; Sigma-Aldrich; A6907 at 1:3,000 dilution. 4. anti-human IgG1 antibody: mouse anti-human IgG1 Fc-HRP; Southern Biotech; 9054–05 at 1:3,000 dilution. 5. anti-human IgG2 antibody: mouse anti-human IgG2 Fc-HRP; Southern Biotech; 9060–05 at 1:3,000 dilution. 6. anti-human IgG3 antibody: mouse anti-human IgG3 Hinge-HRP; Southern Biotech; 9210–05 at 1:3,000 dilution. 7. anti-human IgG4 antibody: mouse anti-human IgG4 Fc-HRP; Southern Biotech; 9200–05 at 1:3,000 dilution), all of them diluted in sample buffer at 3 μL per well dispensed on Biotek Multiflo FX. The incubation of the secondary antibody for 1 h at RT was followed by three washes with PBS-T, the addition of 3 μL per well of Tetramethylbenzidine (TMB) substrate solution with a Fritz Gyger Certus Flex dispenser, incubation of 3 min at RT, and the addition of 3 μL per well 0.5 M H_2_SO_4_ using Fritz Gyger Certus Flex. The plates were centrifuged in the Agilent automated microplate centrifuge after all dispensing steps, except for the addition of TMB. The absorbance at 450 nm was measured in a plate reader (Perkin-Elmer; EnVision), and the inflection points of the sigmoidal binding curves [i.e., the p(EC_50_) values of the respective sample dilution; p(EC_50_) is the negative logarithm of one-half the maximal concentration (EC_50_)] were determined using a custom-designed fitting algorithm (Emmenegger et al., 2020), with plateau and baseline inferred from the respective positive and negative controls in a platewise manner. Negative p(EC_50_) values, reflecting nonreactive samples, were rescaled as zero.

For quality testing, the same procedure was applied as above using the same clone (HP6025) of the HRP-linked secondary antibody but from a different vendor, including a different storage buffer: mouse anti-human IgG4; Invitrogen; A-10654 at 1:500 dilution.

#### Specificity assessment of secondary antibodies

To determine the specificity and the appropriate concentration of secondary antibodies used in this study for immunoglobulin iso- and subtyping, a slightly modified version of the TRABI ELISA protocol rendered above was used. High-binding 384-well plates (Perkin Elmer; SpectraPlate 384 HB) were coated with 20 μL of the antigens listed here and detailed in Key resources table at a concentration of 2.5 μg/mL: 1. IgA purified from human serum (Sigma-Aldrich, 14036), 2. IgM purified from human serum (Sigma-Aldrich, 18260), 3. recombinant monoclonal human IgG1 (Mabylon AG, Switzerland), 4. recombinant monoclonal human IgG2 (EMD Millipore; AG504), 5. recombinant monoclonal human IgG3 (EMD Millipore; AG506), 6. recombinant monoclonal human IgG4 (Mabylon AG, Switzerland), 7. aequimolar mix of afore-mentioned IgG1, IgG2, IgG3, IgG4. Following an incubation of 1 h at 37 °C, plates were washed three times with PBS-T using Biotek El406. Plates were blocked with 40 μL of 5% milk in PBS-T for 1 h and the buffer was removed. Incremental serial dilutions of secondary antibodies listed in the previous chapter and in Key resources table were performed, starting at 1:500 dilution (last dilution: 1:364,500). After an incubation of 1 h at RT, the wells were washed five times with wash buffer, 20 μL TMB substrate solution was added to the wells, incubated for 5 min at RT, and 20 μL per well 0.5 M H_2_SO_4_ was added. The absorbance at 450 nm was measured in a plate reader (Perkin-Elmer; EnVision), the binding curves were visualised, and the p(EC_50_) values were determined as shown (Emmenegger et al., 2020). p(EC_50_) values <0 were rescaled to 0.

### Quantification and statistical analysis

When looking at continuous distributions, tests were performed using the compare_means() function of the ggpubr package 0.4.0 in R version 4.2.0. The method chosen was Kruskal-Wallis (method = kruskal.test) with subsequent Wilcoxon rank sum test (method = wilcox.test) with Holm correction for multiple comparisons, comparing groups with *α* < 0.01 for Kruskal-Wallis against all other groups. Comparisons where *α* < 0.01 with Wilcoxon rank sum test were annotated. Fisher’s test was conducted in Graph Pad Prism, with *α* < 0.01.

Principal component analysis was performed using the prcomp() function as a part of the stats (version 4.2.0) package in R, with center = TRUE und scale = FALSE. The data was then visualised using fviz_pca_biplot() from the factoextra library. The correlation matrix was computed using the cor() function (part of the stats (version 4.2.0) package) in R and visualised as a correlogram using the corrplot() function in the corrplot package in R. The p-values of the correlations were computed using the cor.test() function (part of the stats (version 4.2.0) package) for the Pearson correlation coefficient and *α* < 0.01 was chosen for significance.

For visualisation of individual data points in boxplots, violin plots, ridge plots (ggridges package), density plots (with geom_density_2d where a 2D kernel density estimation was performed on the X and Y coordinates of the input data and the results were displayed with contours), heatmaps (using heatmap.2, a part of the gplots 3.1.1 library), and as scatter dot plots, ggplot2 (version 3.3.5) functions were used. Regression lines and 95% confidence intervals were calculated in ggplot2 and regression coefficients were computed using the stat_cor() function a part of the ggpbubr (version 0.4.0) package. Radar plots were generated with the fmsb package (version 0.7.3) in R.

The fitting was performed as demonstrated (Emmenegger et al., 2020) and the fits were visualised using the matplotlib library in *Python* 3.5.

## Data Availability

• All data underlying this study will be made available for further studies and for comparison with future cohorts by the corresponding authors. • No new code has been developed for this study. • Any additional information required to reanalyse the data reported in this paper is available from the lead contact upon request.
