# Supplementary material for: Both COVID-19 infection and vaccination induce high-affinity cross-clade responses to SARS-CoV-2 variants
Source: iScience. 2022 Jul 16;25(8):104766. doi: 10.1016/j.isci.2022.104766 (PMC9288251; doi:10.1016/j.isci.2022.104766)
Supplement: Document S1. Figures S1–S7 and Table S1 [file mmc1.pdf]

iScience, Volume *25*

Supplemental information

# Both COVID-19 infection and vaccination induce high-affinity cross-clade responses to SARS-CoV-2 variants

Marc Emmenegger, Sebastian Fiedler, Silvio D. Brugger, Sean R.A. Devenish, Alexey S. Morgunov, Alison Ilsley, Francesco Ricci, Anisa Y. Malik, Thomas Scheier, Leyla Batkitar, Lidia Madrigal, Marco Rossi, Georg Meisl, Andrew K. Lynn, Lanja Saleh, Arnold von Eckardstein, Tuomas P.J. Knowles, and Adriano Aguzzi

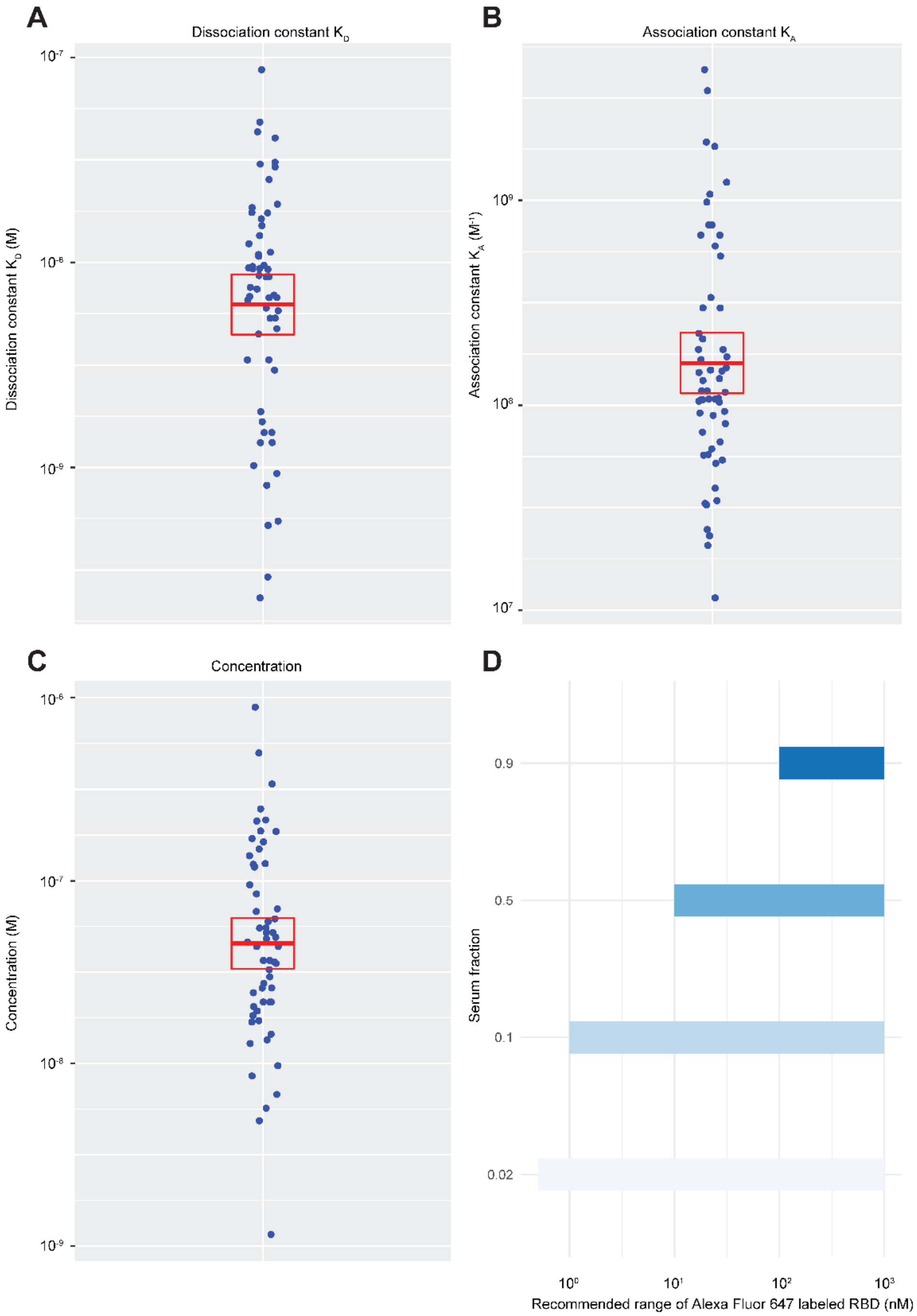

Figure S1. **MAAP sensitivity data for Alexa647-labeled RBD antigen, related to Figs. 2 and 3**. Using a dataset of COVID-19 convalescent donors characterised previously (Fiedler *et al.*, 2022), we have assessed the expected range of affinity and concentration. **A**. and **B**. Affinity expressed as K_D (A) or K_A (B) showed that the limit of detection of the assay lies at approximately 100 nM (K_D), i.e. at 0.01 nM^{-1} (K_A). **C**. Concentrations measured ranged between approximately 1-1000 nM. While higher concentrations can be quantified in this system, concentrations lower than 1 nM likely present the limit of detection of the current assay. **D**. Intrinsic sample background fluorescence is modulated by the fraction of the serum or plasma and affects the fluorescence signal measured. High serum concentrations lead to increased background fluorescence, which decreases the possibility of utilizing lower concentrations of Alexa 647-labeled RBD antigen. A-C: Shown in red are the means with 95% confidence intervals.

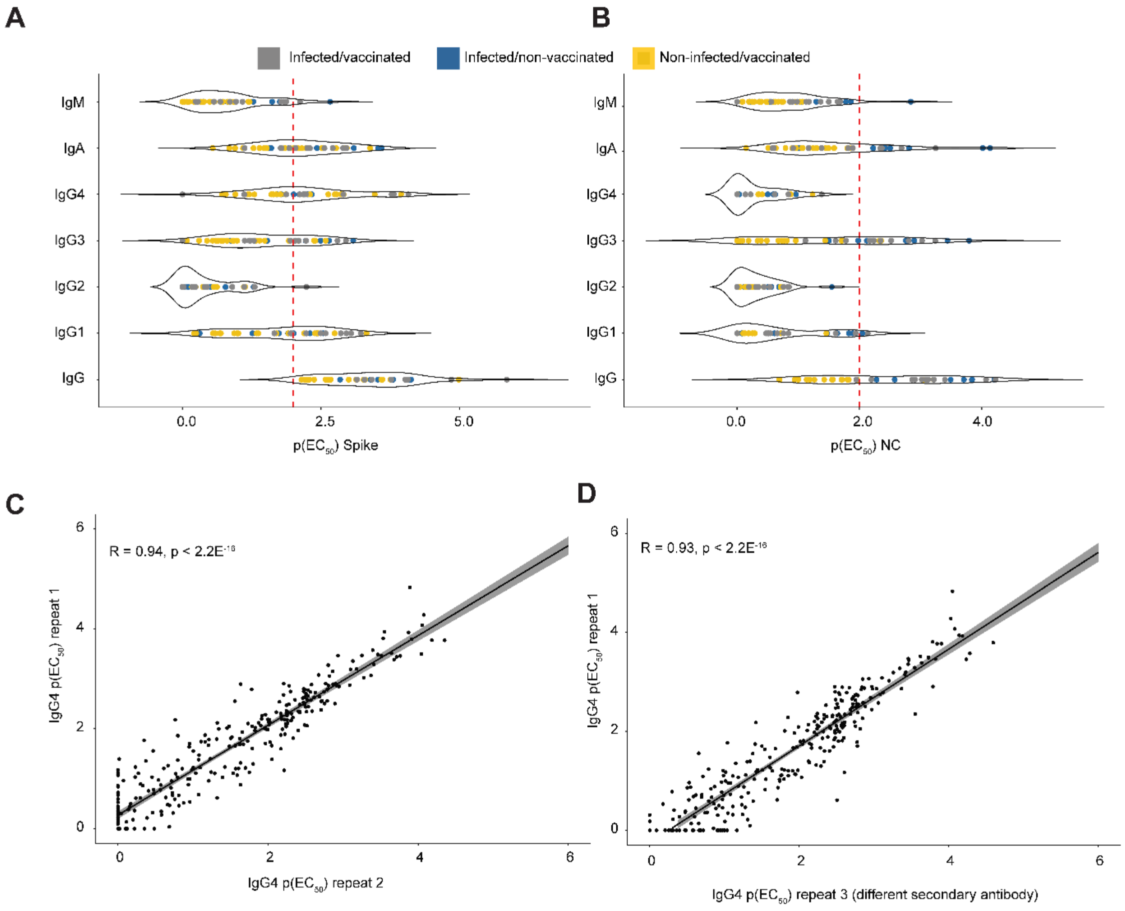

Figure S2. Reactivity profile for Spike and NC and correlations between three repeat IgG4 measurements, using two different secondary antibodies, related to Fig. 4. A. Violin plot showing the reactivity profile for spike ECD. All IgG subtypes contribute to the reactivity, except IgG2. IgM are generally low. **B**. Violin plot showing the reactivity profile for NC protein. The dominant subtype is IgG3. (A) and (B): Infected/vaccinated patients are in grey, infected/non-vaccinated patients are in blue, and non-infected/vaccinated patients are in yellow. **C**. p(EC_{50}) values of the first IgG4 measurements versus p(EC_{50}) values of the second IgG4 measurements. The same secondary antibodies were used. Data are pooled for all antigens. **D**. p(EC_{50}) values of the first IgG4 measurements versus p(EC_{50}) values of the third IgG4 measurements. For the third measurement, a different secondary antibody was used. Data are pooled for all antigens.

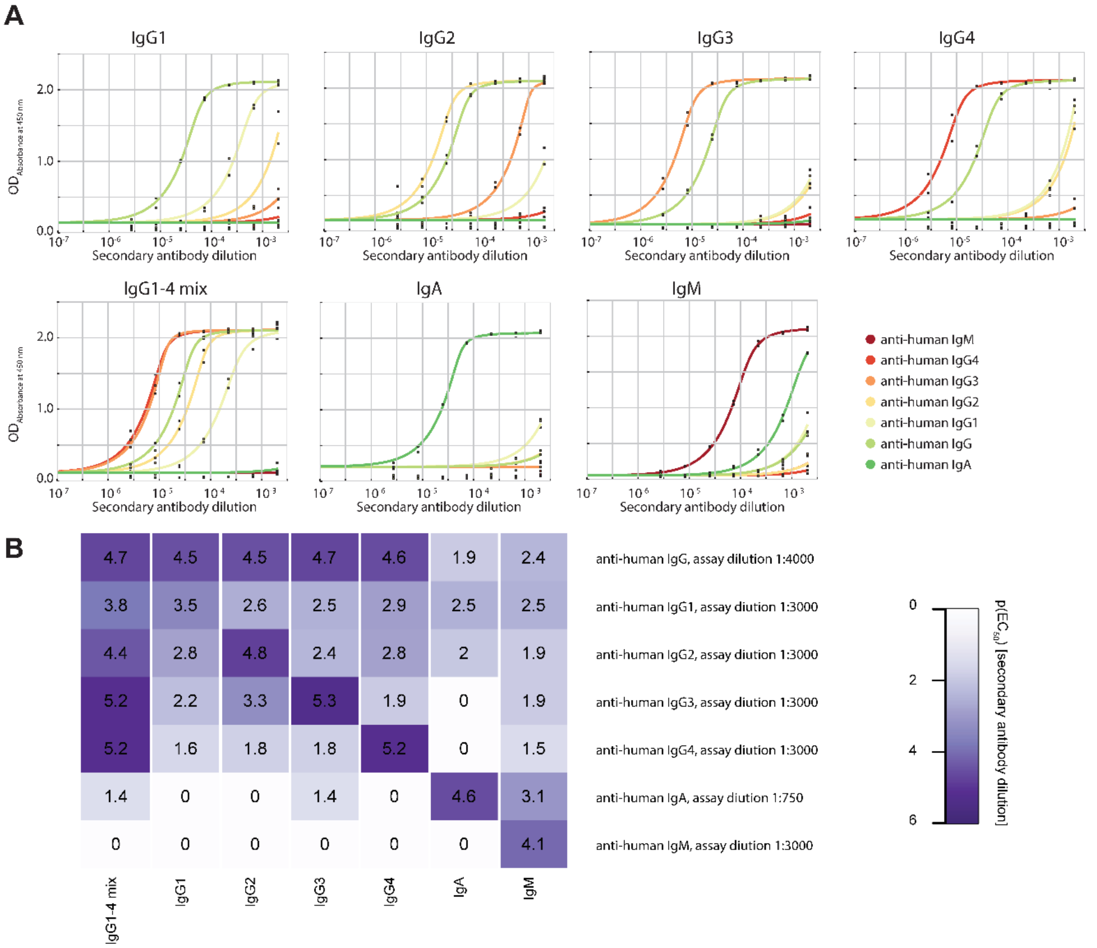

Figure S3. **Assessment of specificity of secondary antibodies used for antibody isotyping and subtyping, related to Figs. 4 and 5**. **A**. Serial dilutions of seven secondary antibodies were tested for specificity and dilution range against purified human serum IgA, purified human serum IgM, recombinant monoclonal IgG1, recombinant monoclonal IgG2, recombinant monoclonal IgG3, recombinant monoclonal IgG4, and against an equimolar mix of all IgG subtypes (IgG1-4 mix). Shown are the respective binding curves of all secondary antibodies tested against above-mentioned antigens, in a serial dilution starting at 1:500 for all antibodies. **B**. Consistent with ELISA-based data shown in this manuscript and with previous work (Emmenegger *et al.*, 2020, 2021), the p(EC_{50}) value was inferred by fitting a logistic regression with plateau values derived from a positive assay control and the baseline from a negative assay control (for detailed methodology and algorithm, see (Emmenegger *et al.*, 2020)) and was then plotted in a heatmap. A and B. It is evident from the IgG1-4 mix that anti-human IgG3 and anti-human IgG4 antibodies, followed by anti-human IgG and anti-human IgG2 are stronger binders at a given dilution than anti-human IgG1. To obtain the best sensitivity with the anti-human IgG1 antibody, a dilution close to 1:1,000 would be indicated; however, this antibody shows considerable cross reactivity to IgG4 and, to a lesser extent, to IgG2. Increasing its concentration would therefore lead to unspecific recognition of other IgG subtypes. A dilution at 1:3,000 for this anti-human IgG1 antibody, is a good trade-of between sensitivity and specificity.

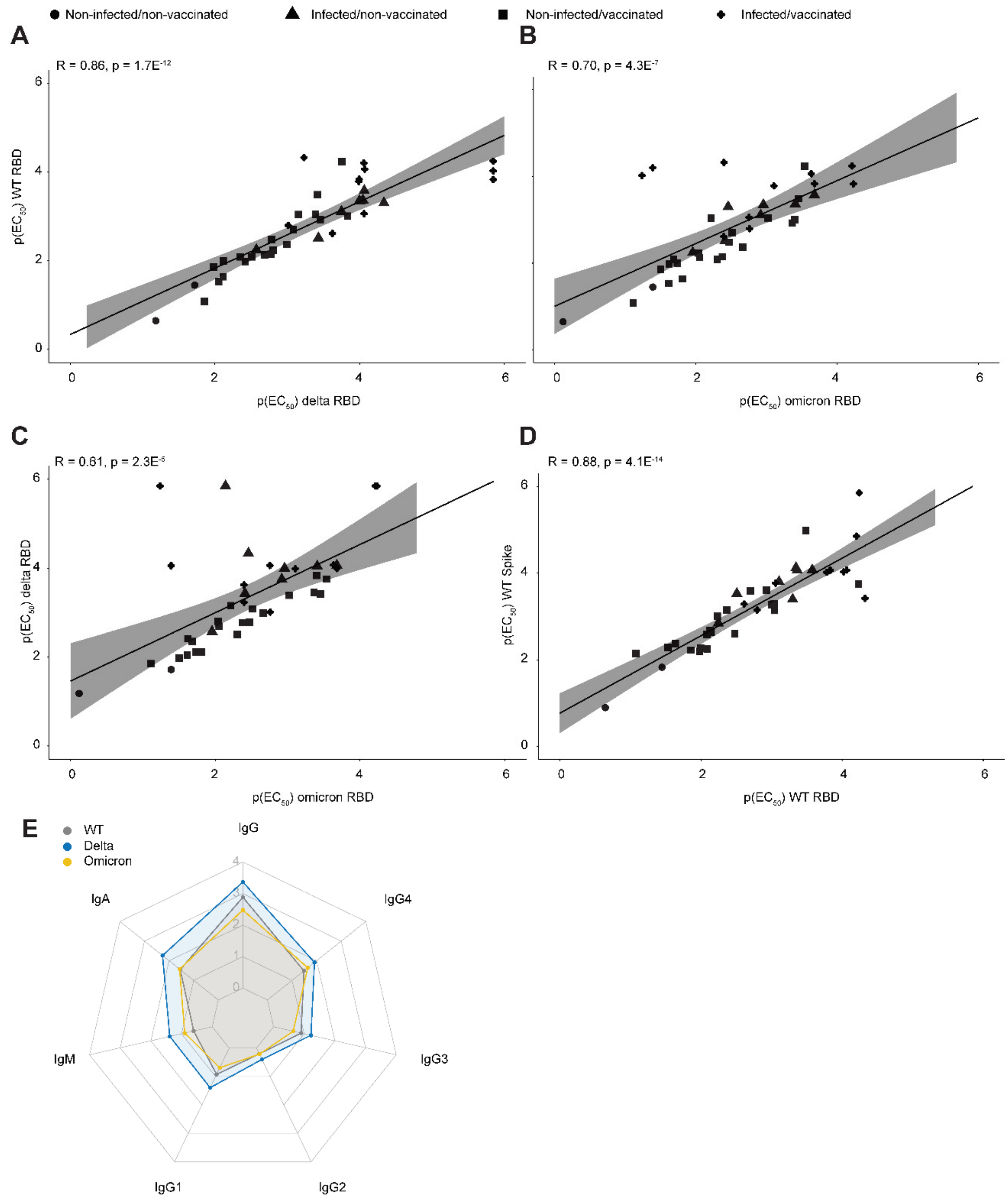

Figure S4. Correlations among RBD variants, the WT spike ectodomain, and comparison of RBD variants, related to Figs. 4 and 5. A. p(EC_{50}) WT RBD versus p(EC_{50}) delta RBD. **B.** p(EC_{50}) WT RBD versus p(EC_{50}) omicron RBD. **C.** p(EC_{50}) delta RBD versus p(EC_{50}) omicron RBD. **D.** p(EC_{50}) WT spike ectodomain versus p(EC_{50}) WT RBD. Shapes indicate the different patient groups. **E.** Mean p(EC_{50}) values of all immunoglobulin iso- and subtypes for WT (grey), delta (blue), and omicron (yellow) RBD variants are shown in a radar plot.

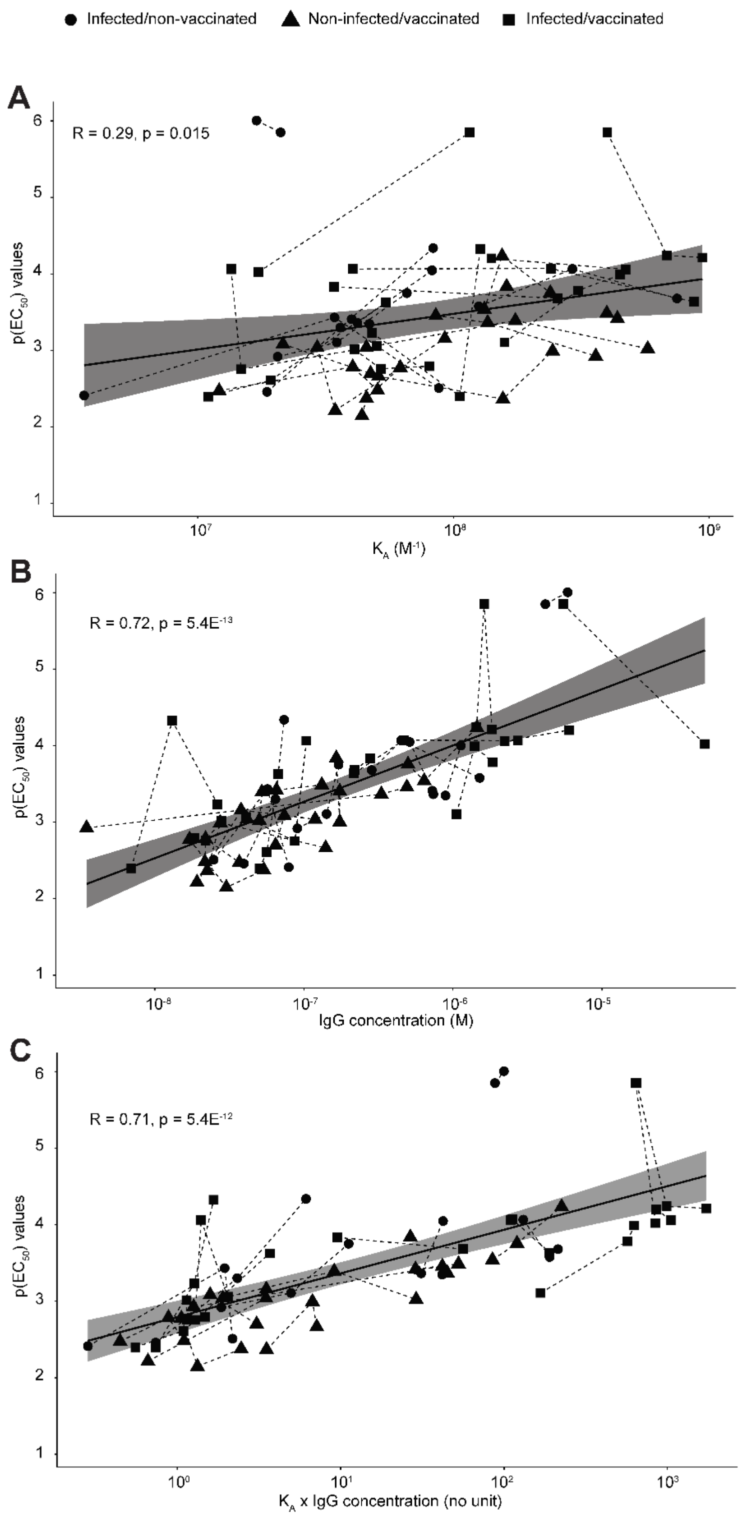

Figure S5. **Correlation of MAAP data with ELISA titers, related to Fig. 4B**. K_A (A), IgG concentration (B), and the product of K_A x IgG concentration (C) were plotted against the respective ELISA p(EC_{50}) values obtained for WT, delta, and omicron RBD variants. The shapes indicate the respective patient groups, as is shown in the main figure.

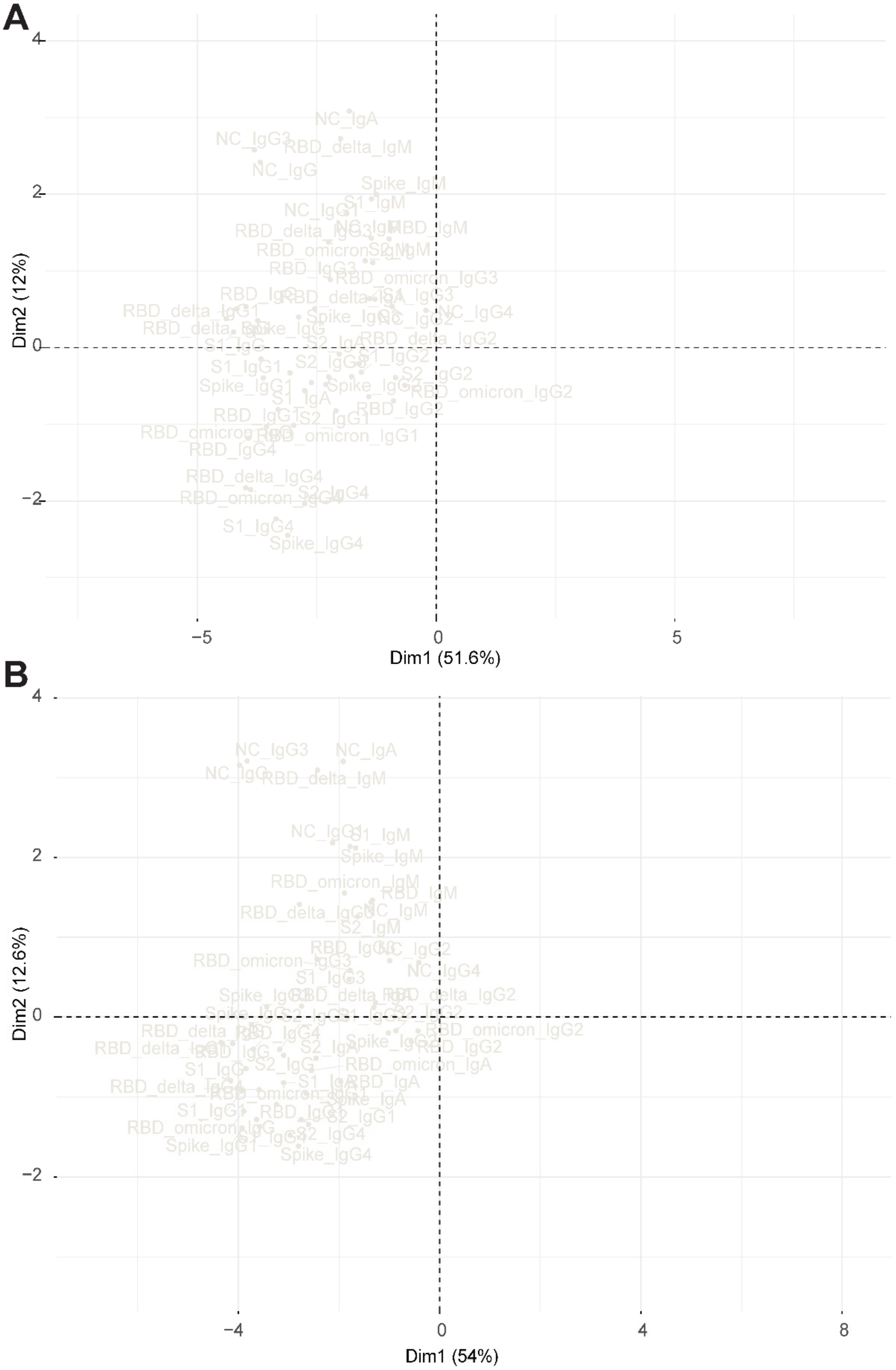

Figure S6. **Variables of PCA shown in main figure, related to Fig. 4C**. The variables of the PCA representations from the main figures are displayed in detail. **A**. PCA including REGN-COV-treated patients. **B**. PCA excluding REGN-COV-treated patients.

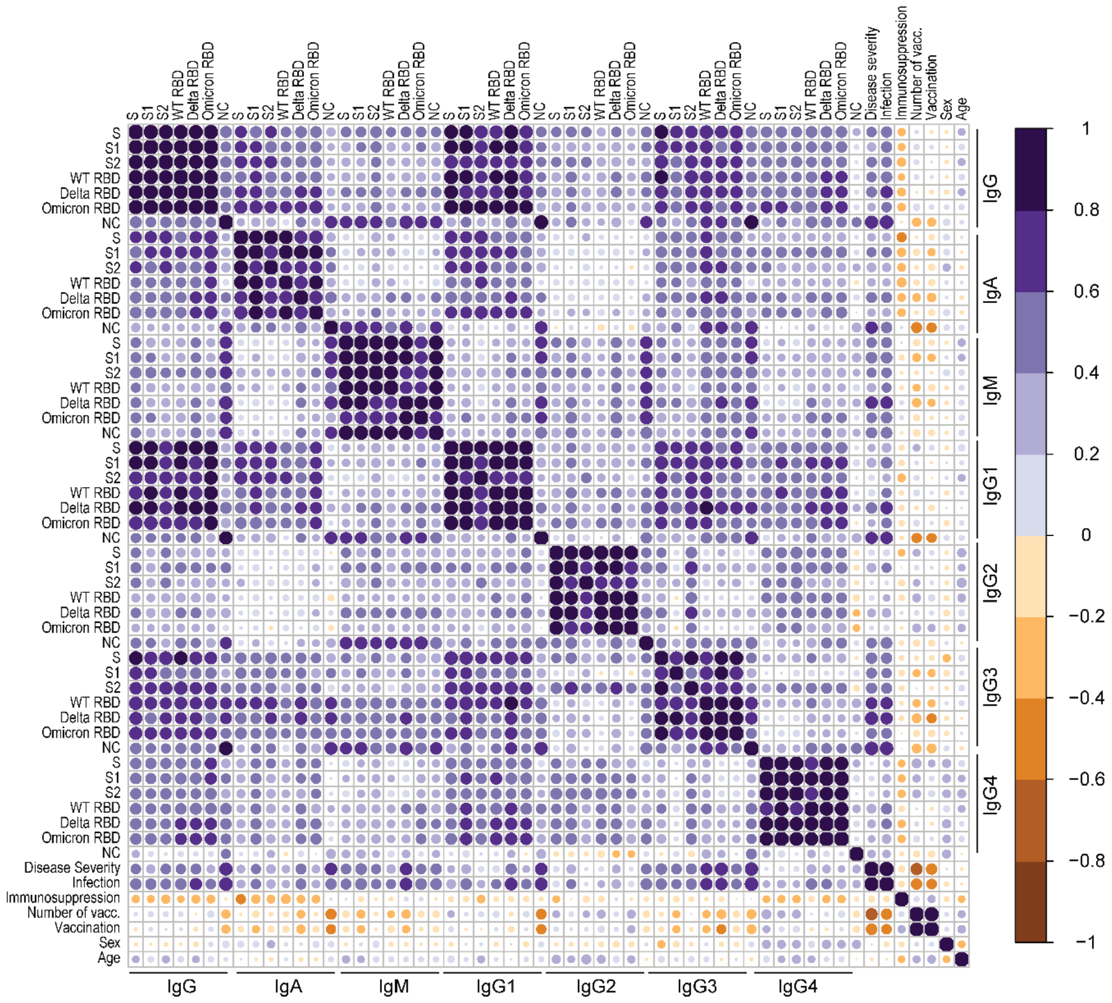

Figure S7. **Correlogram representation of all correlations without restriction by statistical significance, related to Fig. 5**. Correlogram analysis using the TRABI ELISA values combined with features such as disease severity, immunosuppression, number of vaccinations received, sex, and age.

Groups	Patient number	Sex	Age Group	DPO	DPV	REGN-COV treatment	Number of vaccinations	Disease severity Class	Immunosuppression
Non-infected/non-vaccinated	026	M	51-60	-	-	-	0	No disease	Heavy
	037	F	51-60	-	-	-	0	No disease	None
Infected/non-vaccinated	005	F	71-80	13	-	Yes	0	3	None
	019	F	31-40	25	-	-	0	1	None
	012	M	61-70	17	-	-	0	4	None
	010	F	61-70	29	-	-	0	3	None
	002	M	61-70	7	-	-	0	3	None
	015	M	51-60	>30	-	-	0	4	None
	023	F	51-60	15	-	-	0	2	None
	014	M	41-50	>30	-	-	0	3	None
Non-infected/vaccinated	020	F	61-70	-	11	-	3	No disease	None
	013	M	71-80	-	14	-	3	No disease	None
	021	M	51-60	-	176	-	2	No disease	None
	029	F	71-80	-	48	-	2	No disease	Heavy
	022	M	51-60	-	8	-	3	No disease	None
	042	F	71-80	-	207	-	2	No disease	Light
	040	M	>80	-	8	-	3	No disease	None
	028	M	21-30	-	143	-	2	No disease	Light
	039	M	71-80	-	13	-	3	No disease	None
	027	F	71-80	-	95	-	2	No disease	None
	031	M	61-70	-	294	-	2	No disease	None
	034	F	>80	-	251	-	2	No disease	Light
	043	M	>80	-	232	-	2	No disease	None
	049	F	51-60	-	250	-	2	No disease	None
	018	M	51-60	-	287	-	2	No disease	Heavy
	001	M	51-60	-	104	-	2	No disease	None
	046	M	51-60	-	301	-	2	No disease	Heavy
	041	M	71-80	-	283	-	2	No disease	None
	024	F	41-50	-	93	-	3	No disease	Heavy
	038	F	51-60	-	187	-	2	No disease	Heavy
Infected/vaccinated	004	F	71-80	>30	213	-	2	3	Light
	003	M	>80	>30	32	Yes	3	3	None
	050	M	71-80	9	244	Yes	2	3	Light
	011	M	31-40	22	211	-	2	2	Heavy
	016	M	61-70	10	119	Yes	3	3	Heavy
	017	M	>80	0	297	-	2	1	None
	007	F	>80	>30	10	-	3	1	None
	006	M	41-50	8	196	Yes	2	2	None
	009	F	71-80	18	48	-	1	2	Heavy
	008	M	51-60	6	198	-	2	2	Light
	025	F	>80	11	104	-	1	3	None

Table S1. Characteristics of individuals included in study, related to Fig. 4A and Table 1. Sex: male (M) or female (f). Age was partitioned into age groups. DPO = day post onset of COVID-19 symptoms. DPV = day post most recent vaccination. Disease severity class: 0=No disease. 1=Anosmia, fever, fatigue, or headache but did not require hospitalization. 2=Hospitalization without requiring oxygen supplementation. 3=Hospitalization requiring oxygen supplementation. 4=Hospitalization with treatment in the intensive care unit (ICU), mostly including ventilation.
